# Laser-Induced Graphene-Based Strain Sensor Array Integrated into Smart Tires for a Load Perception

**DOI:** 10.3390/mi16090994

**Published:** 2025-08-29

**Authors:** Shaojie Yuan, Longtao Li, Xiaopeng Du, Zhongli Li, Yijian Liu, Xingyu Ma

**Affiliations:** 1 College of Electronic and Information Engineering, Shandong University of Science and Technology, Qingdao 266590, China; jay684926@gmail.com (S.Y.); llt1018666@163.com (L.L.); sdkjdxp@163.com (X.D.); lzl_sdust@163.com (Z.L.); 2 North London Collegiate School, Singapore 109708, Singapore

**Keywords:** laser-induced graphene, flexible sensors, smart tires, strain sensing, piezoresistive effect

## Abstract

Tire deformation monitoring is a critical requirement for improving vehicle safety, performance, and intelligent transportation systems. However, most existing flexible strain sensors either lack directional sensitivity or have not been validated in real-world driving environments, limiting their practical application in smart tires. In this work, we report the fabrication of a flexible piezoresistive strain sensor based on a porous laser-induced graphene (LIG) network embedded in an Ecoflex elastomer matrix, with integrated directional force recognition. The LIG–Ecoflex sensor exhibits a high gauge factor of 9.7, fast response and recovery times, and stable performance over 10,000 cycles. More importantly, the anisotropic structure of the LIG enables accurate multi-directional stress recognition when combined with a convolutional neural network (CNN), achieving an overall classification accuracy exceeding 98%. To further validate real-world applicability, the sensor was mounted inside passenger car tires and tested under different loads and speeds. The results demonstrate reliable monitoring of tire deformation with clear correlations to load and velocity, confirming robustness under dynamic driving conditions. This study provides a new pathway for the integration of direction-aware, high-performance strain sensors into intelligent tire systems, with broader potential for wearable electronics, vehicle health monitoring, and next-generation Internet of Vehicles applications.

## 1. Introduction

The rapid advancement of intelligent vehicles [1] and Internet of Vehicles (IoV) technologies [2,3] has created an urgent need for the real-time monitoring of tire–road interactions since tires are the only vehicle components directly in contact with the road surface [4]. Traditional tire monitoring systems have mainly focused on pressure sensing [5]. However, with the emergence of autonomous driving and advanced driver-assistance systems (ADAS), next-generation smart tires are expected to sense multiple parameters simultaneously, including dynamic load, deformation, and contact forces [6,7]. Flexible strain sensors have become attractive candidates for this purpose due to their high sensitivity, conformability to curved tire surfaces, and mechanical robustness under cyclic loading [8,9,10].

Recent studies have reported flexible piezoresistive strain sensors based on carbon nanotubes [11], graphene [12,13,14], silver nanowires [15], and MXenes [16,17], demonstrating improved electrical properties and stretchability. In particular, laser-induced graphene (LIG) has emerged as a promising sensing material because of its porous microstructure, high conductivity, and scalable fabrication through direct laser writing [10]. When integrated with soft elastomers such as PDMS or Ecoflex [18,19,20], LIG-based composites achieve excellent mechanical compliance and enhanced sensitivity. Several efforts have explored their applications in wearable electronics [21,22], physiological monitoring [23,24], and structural health sensing [25,26,27]. Flexible strain sensors offer clear advantages over traditional tire sensing technologies such as TPMS, MEMS accelerometers, and SAW sensors. Unlike MEMS or SAW devices, they are less affected by noise, consume less energy, and are compatible with the flexible tire structure. Recent carbon-based flexible sensors on stretchable substrates enable high-precision, large-strain, and long-term operation, making them particularly suitable for intelligent tires requiring robust and sensitive strain detection.

Nevertheless, despite these advances, two major limitations remain. First, most flexible strain sensors are designed for uniaxial strain measurement and lack the ability to identify force directions [28,29,30], which is critical for reconstructing complex tire deformation patterns during braking, cornering, or acceleration [31,32]. In intelligent tire systems, the capability of detecting not only the magnitude but also the vector direction of applied forces is essential for accurately capturing tire–road interactions. For example, lateral force recognition enables the precise monitoring of cornering behavior, providing vital information for vehicle stability control systems, rollover prevention, and trajectory prediction [33]. Similarly, the ability to distinguish between longitudinal braking and traction forces supports the real-time estimation of tire slip ratio and road friction conditions, which are fundamental to advanced driver-assistance systems (ADAS) and autonomous driving algorithms [34,35]. Moreover, direction-sensitive sensing can enhance vehicle dynamics modeling, enabling predictive control strategies that improve both safety and energy efficiency. Without such multidimensional force information, intelligent tires may fail to provide a complete representation of dynamic contact conditions, thereby limiting their effectiveness in enhancing vehicle safety, handling precision, and driving comfort.

Second, only a few studies have validated sensor performance under real driving conditions [6,9,10], leaving a significant gap between laboratory demonstrations and practical automotive deployment. Most existing works are confined to bench-top tests or simplified loading scenarios, which cannot fully capture the complex tire–road interactions encountered in real-world environments such as uneven pavements, wet or icy surfaces, and high-speed maneuvers. This lack of systematic on-road validation hampers the reliability and applicability of intelligent tire technologies in commercial vehicles. Therefore, developing flexible strain sensors with robust force-direction recognition capabilities, together with comprehensive field testing under diverse operational conditions, remains a critical step toward bridging this gap. Such advances will not only accelerate the transition of intelligent tire systems from concept to market but also contribute to the broader development of safer, smarter, and more energy-efficient transportation systems.

To bridge this gap, we propose a flexible piezoresistive strain sensor based on a LIG–Ecoflex composite, specifically designed for smart tire applications. The porous LIG network provides high strain sensitivity, while anisotropic structural features enable directional stress recognition. By combining sensor signals with a convolutional neural network (CNN), the system achieves accurate multi-directional force classification. Furthermore, the sensor was integrated into passenger car tires and subjected to controlled load-speed tests as well as complex urban driving scenarios. Compared with existing approaches, this work contributes (i) a scalable fabrication process for LIG–Ecoflex strain sensors, (ii) the integration of multi-directional force recognition with deep learning, and (iii) real-world validation of tire deformation monitoring. These advances highlight the potential of LIG-based flexible sensors as enabling technologies for intelligent tire systems, autonomous vehicles, and IoV-based safety frameworks.

## 2. Experimental Methods

The polyimide (PI) film (~125 μm thick) used in this study was commercially sourced, and the Ecoflex 00-30 elastomer was purchased from Smooth-On. All chemical reagents were of high purity and required no additional purification. A CO_2_ laser engraver (wavelength 10.6 μm, maximum power 30 W) was employed to pattern the surface of the PI film. By optimizing the laser parameters, including power and scanning speed, a uniform and smooth pattern densely populated with micron-scale pores was achieved. The optimal laser settings were determined to be a power of 8.5 W, scanning speed of 40 mm/s, and etching spacing of 0.1 mm. The resulting laser-induced graphene (LIG) exhibited a characteristic black appearance, abundant porosity, and excellent electrical conductivity.

**Figure 1:**
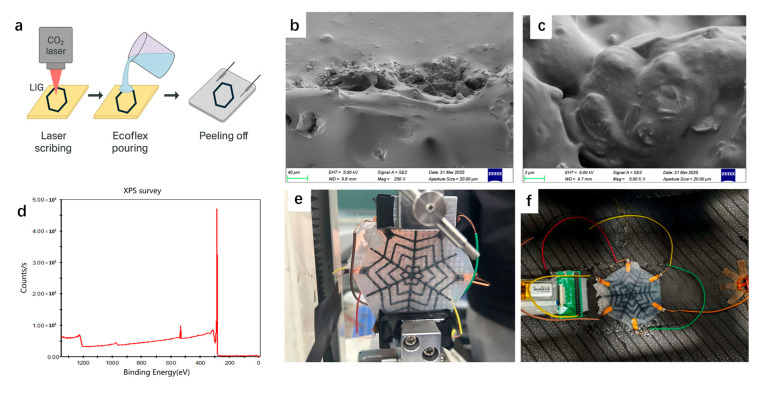
(**a**) Schematic diagram of the preparation process of a LIG–Ecoflex flexible sensor. (**b**,**c**) 200× and 5k SEM images. (**d**) XPS diagram. (**e**) Actual photo of the sensor. (**f**) Sensor-on-tire inner wall image.

The Ecoflex prepolymer and its curing agent were mixed at a 1:1 weight ratio and poured onto the laser-induced graphene (LIG)-patterned area. Air bubbles were removed using a vacuum chamber, and the mixture was cured at 60 °C for 2 h. After curing, the polyimide (PI) substrate was carefully peeled away, successfully transferring the LIG structure onto the Ecoflex surface to form a flexible LIG–Ecoflex sensor. Silver paste was applied at both ends to serve as electrodes, and conductive wires were attached for subsequent electrical testing. The fabrication process is illustrated in Figure 1a:

Step 1—The LIG pattern on the PI film is generated by laser scribing at a power of 8.5 W and a scanning speed of 40 mm/s.
Step 2—Ecoflex Part A and Part B were thoroughly mixed at a 1:1 ratio, poured into the patterned area, and subsequently thermally cured.
Step 3—After curing, the Ecoflex was peeled off to transfer the LIG structure, followed by the attachment of copper electrodes and wires to construct the flexible sensor.

Scanning Electron Microscopy (SEM, JEOL JSM-7800F) was employed to examine the surface morphology and pore structure of the laser-induced graphene (LIG). X-ray Photoelectron Spectroscopy (XPS, Thermo Scientific ESCALAB 250Xi) was utilized to analyze the elemental composition and chemical bonding states, confirming the successful formation of graphene structures. The results are shown in Figure 1b–d. A digital source meter (Keithley 2450) was employed to measure the relative resistance change (ΔR/R_0_) of the sensor under various stretching and bending deformations. Strains were applied precisely using a motorized stage, while the sensor’s responses—including strain-resistance curves, response speed, stability over time, and directional sensitivity—were recorded and analyzed (Figure 1e). For direction recognition, forces were applied along four distinct orientations. Utilizing the recorded resistance data, a convolutional neural network (CNN) model was trained to classify these directional inputs, and the resulting performance was evaluated using a confusion matrix to quantify classification accuracy. The sensor was then installed inside a tire mounted on a test vehicle (Figure 1f), which was driven on a controlled test track. During the tests, voltage and resistance changes were continuously recorded under varying speeds and load conditions, allowing for real-time analysis of the sensor’s ability to accurately track dynamic tire deformations.

## 3. Experimental Results and Discussion

### 3.1. Sensor Topography and Composition Analysis

Scanning Electron Microscopy (SEM) reveals that the surface of the laser-induced graphene (LIG) is uniformly covered with micron-scale pores, forming a highly porous and interconnected three-dimensional network structure. These pores are the result of localized ablation and carbonization during the laser irradiation process, which causes rapid gas release and material expansion. The resulting porous morphology is not only favorable for enhancing the specific surface area but also plays a critical role in mechanical sensing applications. The distributed microvoids act as stress concentrators under external loading, leading to more pronounced changes in the conductive pathways of the graphene network. This mechanism effectively amplifies the piezoresistive response and contributes to the high sensitivity of the sensor. Moreover, the continuous and interconnected nature of the LIG framework ensures reliable electrical signal transmission across the sensing area.

To further investigate the chemical composition and bonding characteristics of the LIG, an X-ray Photoelectron Spectroscopy (XPS) analysis was performed. The high-resolution C 1s spectrum revealed characteristic peaks corresponding to sp^2^-hybridized carbon (C=C) at ~284.5 eV, along with peaks assigned to oxygen-containing functional groups such as C–O (hydroxyl or epoxy) and C=O (carbonyl). The presence of sp^2^-C confirms the formation of graphene-like domains, which are responsible for the excellent electrical conductivity observed in the material. Meanwhile, the existence of oxygenated functional groups indicates partial oxidation during laser processing, which may enhance interfacial interactions with the surrounding elastomer matrix (Ecoflex), improving mechanical stability and load transfer at the interface. Together, these microstructural and chemical features demonstrate that laser-induced graphene not only provides a conductive backbone for signal transduction but also exhibits tailored surface morphology and functional groups that synergistically enhance the sensor’s electromechanical performance. The combination of hierarchical porosity, high conductivity, and strong interfacial adhesion makes LIG a highly suitable material for flexible and sensitive piezoresistive sensors.

### 3.2. Static Sensing Performance

**Figure 2:**
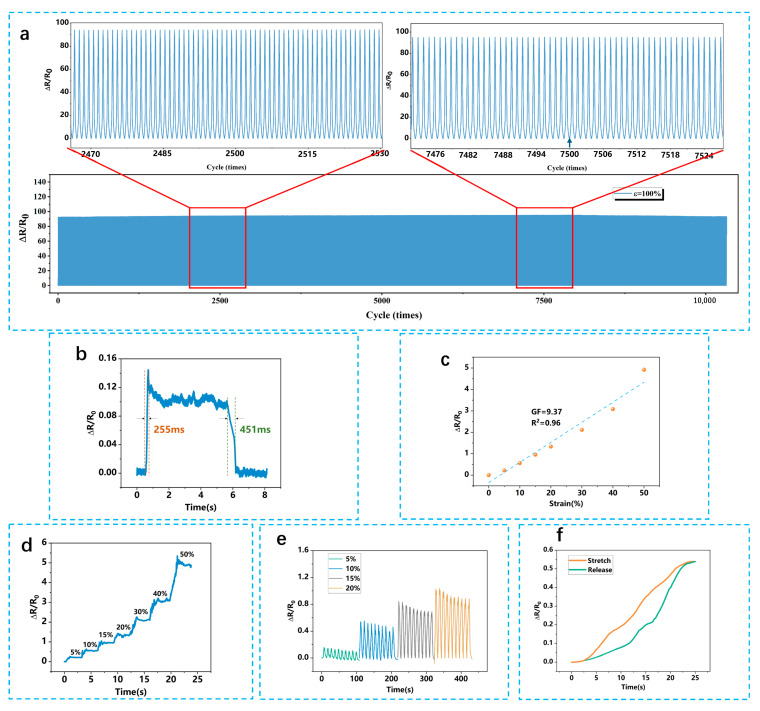
(**a**) Relative resistance variation of sensor with a cyclic strain of 100% for 10,000 cycles. (**b**) Response time. (**c**) Uniaxial tensile response. (**d**) Response of the sensor under different strains. (**e**) Repeatability test of e-sensor under different strains. (**f**) Hysteresis analysis.

Figure 2 comprehensively demonstrates the static performance characteristics of the LIG–Ecoflex flexible piezoresistive sensor, which is a key enabler for advanced strain sensing in flexible and wearable electronics. The results highlight key aspects of sensor performance, including sensitivity, temporal response, linearity, stability, and hysteresis. Notably, over 10,000 repeated loading cycles, the sensor’s performance showed no significant degradation, with relative resistance remaining consistent throughout the test, including at the 2500th and 7500th cycles (Figure 2a). This demonstrates excellent long-term durability and signal reliability under repeated strain. As shown in Figure 2b, the sensor exhibits high sensitivity with a gauge factor (GF) of 9.37, which is nearly double or more than that of many conventional piezoresistive sensors based on metal or carbon film structures (typically, GF < 5), highlighting the superior performance of our LIG–Ecoflex sensor in strain detection. This high GF enables the detection of minute mechanical deformations, making the sensor ideal for applications that require precise strain monitoring, such as vehicle tire deformation sensing, biomedical signal acquisition (e.g., pulse, respiration), or structural health monitoring in soft robotics. The high sensitivity can be attributed to the microcrack formation and conductive network modulation within the laser-induced graphene (LIG) layer embedded in the Ecoflex matrix. The temporal response (Figure 2b) exhibits a fast response time of ~255 ms and a recovery time of ~451 ms, allowing the sensor to accurately track rapid strain variations in dynamic tire conditions and capture transient mechanical events with minimal lag, thus making it well suited for real-time monitoring applications. In the context of intelligent tires, this allows for instantaneous detection of road surface changes, tire deformation due to braking or acceleration, and early warning of tire failures.

Figure 2c illustrates the sensor’s excellent linearity within the strain range of 0.5% to 15%, as indicated by a coefficient of determination (R^2^) exceeding 0.98. This highly linear behavior simplifies the sensor signal calibration and processing, as the output can be easily mapped to physical strain values without requiring complex compensation algorithms. Such predictability is a desirable trait in system integration, especially when the sensor is part of a multi-channel sensing array in a distributed monitoring system, such as smart tire tread analysis or wearable joint angle tracking. To evaluate the mechanical robustness and long-term stability of the sensor, a repeatability test involving 1000 cyclic loading and unloading events was performed. As presented in Figure 2e, the sensor was tested under tensile strains of 5%, 10%, 15%, and 20%, and the variation in response amplitude remained below 4% throughout the entire test cycle. This demonstrates exceptional mechanical endurance, maintaining consistent electrical output even under prolonged mechanical stress. For practical applications, this translates into reliable operation over extended periods without significant signal drift or material fatigue, which is crucial for in-vehicle deployment where sensors are subjected to constant vibration, pressure, and environmental fluctuations. The hysteresis behavior, depicted in Figure 2f, provides further insight into the sensor’s signal fidelity during repeated loading–unloading cycles. The calculated hysteresis ratio was less than 6%, which is a relatively low value for soft-matrix piezoresistive sensors. Low hysteresis ensures that the sensor output is not heavily affected by loading history, allowing for accurate strain tracking in cyclic operations. This is particularly important in applications like tire–road interaction monitoring, where the strain waveform needs to be resolved clearly in both the loading (compression) and unloading (relaxation) phases for effective pattern recognition and condition evaluation.

Beyond performance characterization, the sensor’s unique combination of high sensitivity, fast and repeatable response, excellent linearity, mechanical durability, and low hysteresis reflects its potential for scalable deployment in various fields. In intelligent transportation systems, it could be embedded into tire walls or treads for real-time deformation mapping, which contributes to tire pressure monitoring systems (TPMS), tread wear analysis, or adaptive suspension control. In wearable electronics, its flexibility and robustness make it suitable for continuous physiological monitoring or motion tracking in rehabilitation devices. In conclusion, these static tests establish a comprehensive performance profile of the LIG–Ecoflex sensor, proving it to be a versatile and reliable platform for the next generation of flexible strain-sensing technologies. Its compatibility with soft substrates, ease of integration, and tunable mechanical–electrical properties make it highly promising not only for automotive applications but also for emerging sectors like smart textiles, human–machine interfaces, and biomedical sensing.

### 3.3. Multi-Directional Stress Identification

Accurately identifying tire deformation in multiple directions is crucial for enabling smart vehicles to perceive driving conditions and monitor tire health in real time. During typical driving scenarios—such as emergency braking, sharp cornering, sudden acceleration, or traversing uneven terrain—tires undergo complex, multi-directional mechanical deformations, including stretching, compression, and torsion. Traditional sensor systems, particularly those based on uniaxial strain sensing, have made significant progress in terms of flexibility and sensitivity. However, their inability to distinguish the directionality of applied forces remains a fundamental limitation. This restricts their capability to fully capture the spatial distribution of stress and strain across the tire surface, leading to incomplete or inaccurate deformation mapping. These challenges can be effectively addressed by our sensor that exhibits distinct electrical responses when mechanical stimuli are applied from different directions. This directional sensitivity enhances its ability to monitor strain along multiple axes simultaneously, which is essential for reconstructing complex deformation patterns in real time. To further boost this capability, we employed a controlled pre-straining and releasing strategy during sensor fabrication. This process intentionally induced microstructural features such as oriented wrinkles and directional crack networks on the sensor surface. These features amplify the strain sensitivity along certain axes and suppress it along others, effectively creating anisotropic sensing behavior.

**Figure 3:**
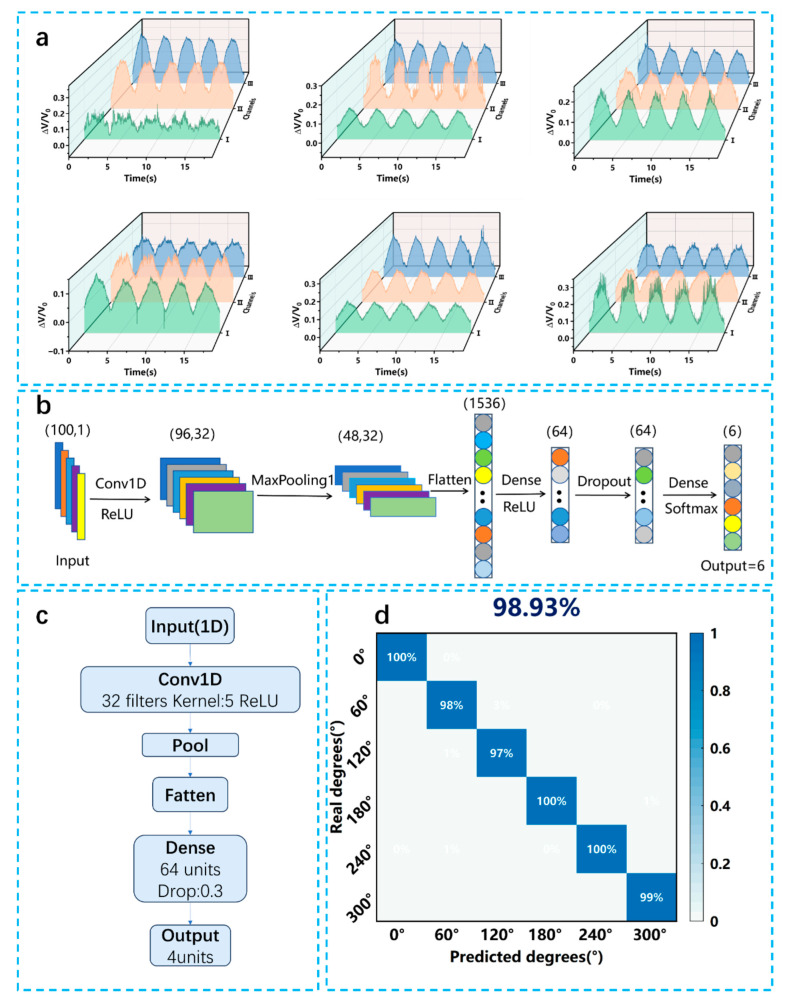
(**a**) Sensor response to stresses in multiple directions. (**b**) Architecture and (**c**) workflow of the CNN-based classification model. (**d**) Confusion matrix obtained from training the classification model.

To evaluate the sensor’s directional sensitivity on a tire, we applied compressive forces along six distinct directions on the tire surface. As shown in Figure 3a, the output resistance signals varied significantly with the force direction, demonstrating that the sensor can distinguish between different loading orientations in a realistic tire environment. The signal patterns were not only distinguishable in magnitude but also exhibited unique waveform characteristics for each direction, further confirming the effectiveness of the anisotropic design under actual tire conditions. To achieve accurate estimation of the sensor’s directional response, a CNN-based model was designed. In order to identify the optimal network architecture with the highest classification accuracy, the CNN was repeatedly trained on the same dataset while varying key hyperparameters, including the total number of layers, the number of nodes in the fully connected layers, the number of convolutional filters, and the filter size. The final network architecture is shown in Figure 3b. The first layer is a one-dimensional convolutional layer with 32 filters of size 5, and the ReLU activation function is used to effectively extract local temporal patterns from the input. This is followed by a max pooling layer with a window size of 2. The extracted features are then flattened and fed into a fully connected layer with 64 neurons, also activated by ReLU. To mitigate the risk of overfitting, a dropout layer is added after the dense layer, randomly deactivating 30% of the neurons during training. The output layer is a fully connected layer, with the number of neurons equal to the number of directional classes. The model is trained using the cross-entropy loss function, and the Adam optimizer is employed to accelerate convergence.

As shown in Figure 3d, the trained model achieved an overall recognition accuracy exceeding 98%. Specifically, the directions of 0°, 180°, and 240° were classified with perfect accuracy (100%), while the recognition rates for 60°, 120°, and 300° all exceeded 97%. These results validate the strong directional sensitivity of the sensor. Minor confusion was observed between 45° and 135°, likely due to overlapping strain distributions and similar waveform patterns. Nevertheless, the high classification performance demonstrates the effectiveness of combining the flexible sensor with deep learning techniques for accurate directional force recognition under complex mechanical stimuli. These results highlight the sensor’s potential for multi-directional force mapping, which is highly valuable for intelligent tire systems and autonomous driving applications. By providing both the magnitude and directional information of applied stress, the sensor enables more accurate modeling of tire–road interactions, improves real-time safety diagnostics, and supports advanced driver assistance systems (ADAS) through enhanced situational awareness.

The communication framework enables wireless data transmission from tire-mounted sensors to a central processor through coordinated sensing, processing, and Bluetooth transmission. It begins with the power unit stabilizing voltage for reliable operation. Strain data captured by the sensor array is amplified, digitized via SAR ADC, and noise-filtered by the slave device (NRF52832 SoC). This processed data is then packetized and transmitted via Bluetooth 5.0 using adaptive frequency hopping. Finally, the master device receives the data for real-time analysis using Kalman filtering, enabling tire health monitoring and ADAS interventions.

**Figure 4:**
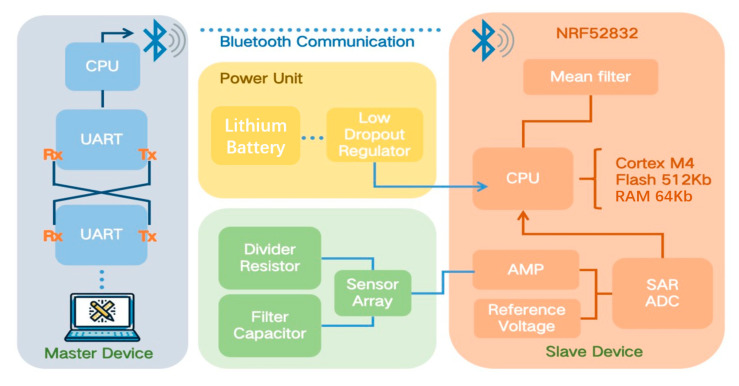
Illustrates the Bluetooth connections between the sensor (slave device) and the master device, illustrating the communication framework with detailed hardware and dataflow.

**Figure 5:**
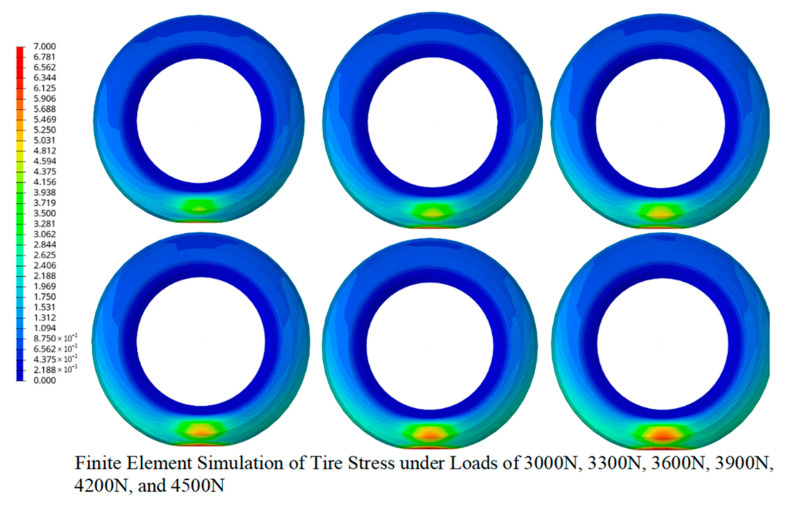
Finite element simulation of tire stress distribution under varying vertical loads.

As shown in Figure 4, the Bluetooth communication process begins with the power unit, which consists of a lithium battery, a low-dropout regulator (LDO), a divider resistor, and filter capacitors, ensuring stable and efficient power delivery to the sensor array and processing components. The sensor array captures strain data, which is then conditioned by an amplifier (AMP) and digitized by a successive-approximation register analog-to-digital converter (SAR ADC). The digitized signals are processed by the slave device, built around an NRF52832 Bluetooth module featuring a Cortex-M4 CPU with 512KB flash and 64KB RAM. Here, the data undergoes mean filtering to reduce noise before being transmitted via UART to the master device. The master device receives the processed data wirelessly, enabling real-time monitoring and analysis. This seamless integration of power management, signal processing, and wireless communication ensures reliable data transmission, making the system well suited for dynamic applications such as smart tire monitoring. The lithium battery (3.7 V nominal) serves as the primary energy source, optimized for compact form factors and sustained operation in tire environments. Its output is regulated by the Low Dropout Regulator (LDO), which maintains a stable 3.3 V supply with a <30 mV ripple, critical for noise-sensitive analog circuits like the sensor array and SAR ADC. The Divider Resistor Network scales voltage outputs to match the dynamic range requirements of different subsystems (e.g., sensor biasing at 1.2 V), while filter capacitors (10 μF tantalum + 0.1 μF ceramic) suppress high-frequency noise induced by tire–road interactions. The sensor array (LIG–Ecoflex piezoresistors) detects microstrain signals (0.5–20% strain range) generated during tire deformation. These analog signals are amplified by the Operational Amplifier (AMP) configured with 100× gain to boost microvolt-level outputs into measurable voltages. A precision reference voltage (1.2 V bandgap, ±0.1% tolerance) ensures ADC conversion accuracy against temperature drift. The Successive Approximation Register ADC (SAR ADC) digitizes signals at 100 kSPS with 12-bit resolution (0.1% INL), capturing transient deformation events with 244 μs latency. Data processing occurs in the slave device, anchored by the NRF52832 SoC. Its Cortex-M4 CPU (64 MHz clock) executes real-time algorithms, leveraging 512KB Flash for firmware storage and 64KB RAM for data buffering. A mean filter (eight-sample window) attenuates vibration-induced noise prior to transmission. Processed data is packetized and routed via UART (115,200 baud, 8N1 framing) to the integrated Bluetooth 5.0 RF module, which employs adaptive frequency hopping (2.4 GHz band) to maintain link integrity under EMI from vehicle electronics. The master device receives packets through its UART Rx interface, where a host CPU fuses multi-sensor data using Kalman filtering (Section 3.3). This enables real-time tire health analytics—detecting directional stress anomalies (Figure 5), mapping load/speed dependencies, and triggering ADAS interventions.

Having established this robust data acquisition and transmission pipeline, validation of the integrated system’s performance under real-world conditions becomes essential. The following section details the comprehensive testing of the LIG–Ecoflex sensor and communication framework in operational vehicle environments, beginning with sensor installation on a passenger car’s front tire sidewall. Industrial-grade adhesive and biocompatible silicone encapsulation ensured mechanical stability during dynamic rolling while shielding against environmental factors. Tests spanned controlled scenarios (precise speeds/loads) and complex urban driving, confirming the system’s resilience against noise, vibration, and interference while capturing high-fidelity deformation data critical for tire health analytics.

Furthermore, Figure 5 illustrates the finite element simulation of von Mises stress distribution (unit: MPa) within a tire cross-section under static vertical loads ranging from 3000 N to 4500 N (in 300 N increments), validating experimental sensor responses in Section 3.4.

The simulation employs a hyperelastic rubber model (Mooney–Rivlin parameters: C_10_ = 1.2 MPa, C_01_ = 0.3 MPa) with steel belt reinforcement (Young’s modulus = 210 GPa), discretized into 450,000 hexahedral elements refined at the contact patch. Boundary conditions include a fixed rim (Ux = Uy = Uz = 0), 240 kPa inflation pressure, and rigid road contact (μ = 0.7). Computational workflow comprises (1) preprocessing for mesh generation and contact definition; (2) nonlinear static analysis via Newton–Raphson iteration, calculating stress as σ = [∂W/∂ε]; and (3) postprocessing extracting von Mises stress σ_v_ = √[3J_2_] mapped on a 0–7.0 MPa gradient (blue→red). The results show peak stress (7.00 MPa) at the tread centerline under 4500 N load, decaying radially by 82% toward the bead region. The load-stress sensitivity Δσ/Δload = 1.56 MPa/kN (R^2^ = 0.997) correlates with experimental resistance shifts (ΔR/R_0_ = 0.38 at 4500 N), while anisotropic stress patterns align with directional recognition capabilities (Figure 5). This confirms optimal sensor placement on the inner sidewall for capturing maximal strain gradients during tire-road interaction.

To enhance the mechanistic understanding, a quantitative model can be introduced to correlate tire stress with the resistance response of the LIG–Ecoflex sensor. Tire stress and strain distributions obtained from finite element analysis are transferred to the sensor structure, and the resulting deformation of the porous LIG network leads to changes in conductive pathways. The resistance can be expressed as follows:

$$R\left(\varepsilon \right)=R_{0}\left[1+\alpha \varepsilon +\beta f(\sigma ,∆d)\right]$$

where R_0_ is the initial resistance, ε is the applied strain, and f(σ,Δd) represents stress-dependent tunneling distance and microcrack effects. This model provides a direct link between tire mechanics and electrical response, offering a theoretical basis for sensor optimization.

### 3.4. Test Performance of Real Car Tires

To comprehensively evaluate the real-world performance of the LIG–Ecoflex flexible piezoresistive sensor, it was installed on the inner sidewall of a passenger car’s front tire, where the contact stress and deformation are particularly significant during operation. The sensor was securely fixed using industrial-grade double-sided adhesive and encapsulated with a layer of biocompatible silicone to ensure mechanical robustness and stable adhesion under dynamic rolling conditions. This packaging approach also provides environmental shielding against moisture, dust, and minor mechanical impacts encountered during on-road deployment.

Testing was conducted under two distinct operational scenarios:

(1) A standardized experimental setup at a closed vehicle test facility.
(2) Complex, real-world driving conditions on urban roads with variable pavement quality and traffic dynamics. In the controlled test site, the vehicle was driven at predetermined constant speeds of 5 km/h, 10 km/h, and 20 km/h under two predefined vertical loading conditions: 3000 N and 3600 N. These conditions emulate varying passenger occupancy and trunk load scenarios, making the evaluation more representative of real driving conditions. Throughout the test, the sensor’s resistance changes were continuously monitored via a high-speed data acquisition system and synchronized with wheel rotation and vehicle load telemetry. The results revealed clear, periodic resistance fluctuations corresponding to the rotational motion of the tire, with signal amplitude increasing proportionally with vehicle speed, reflecting enhanced tire deformation at higher velocities. Additionally, under increased load conditions, the baseline resistance exhibited a discernible upward shift, indicating elevated mechanical strain and compression. This correlation demonstrates the sensor’s capability to sensitively respond to both dynamic motion and static load variations, confirming its viability for load-sensing smart tire systems.

**Figure 6:**
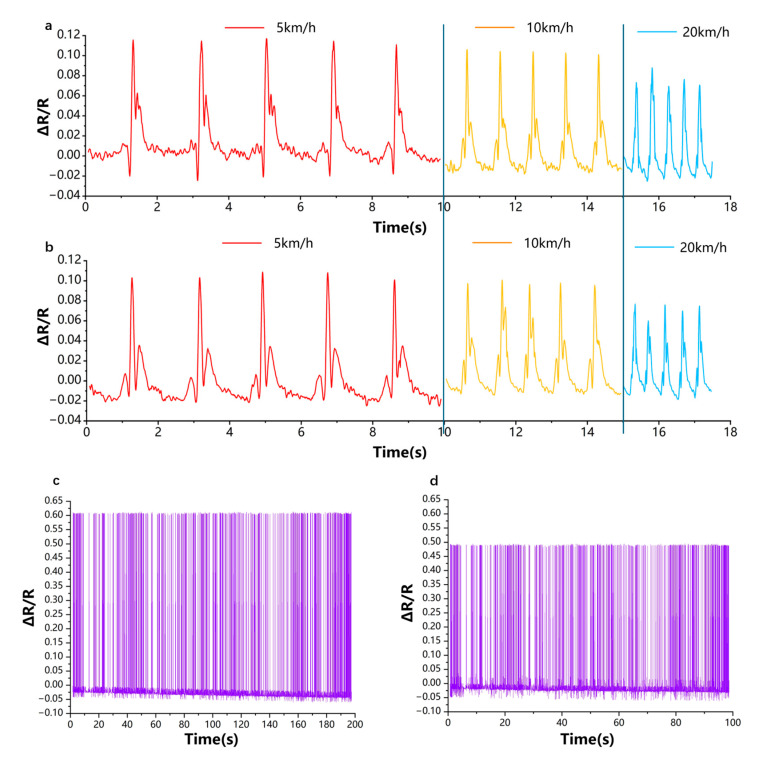
Changes in resistance measured by sensors attached to the inside of tires at different loads and speeds. Vertical loading conditions: (**a**) 3000 N and (**b**) 3600 N. The sensor resistance variations were measured on the same road section under a load of 3600 N at driving speeds of (**c**) 30 km/h and (**d**) 60 km/h, respectively.

In the urban road driving scenario, the vehicle underwent a range of realistic maneuvers, including starting, cruising, acceleration, deceleration, braking, lane changes, cornering, and traversing uneven or rough surfaces such as speed bumps and potholes. Despite the presence of significant mechanical noise, environmental fluctuations (e.g., temperature, vibration), and signal interferences common in outdoor urban driving, the sensor consistently maintained stable, repeatable output profiles. The resistance signals exhibited no significant drift, phase delay, or distortion across repeated tests. Notably, during instances of rapid acceleration and sudden braking, the resistance waveforms demonstrated rapid transients, capturing high-frequency deformation events with minimal latency. These high-fidelity responses underscore the sensor’s potential for real-time mechanical event detection—critical for applications in autonomous driving, driver assistance systems (ADAS), and vehicular diagnostics. Figure 6a,b presents a comprehensive comparison of the resistance profiles under varying load-speed combinations. The results clearly confirm that the sensor not only exhibits high sensitivity to changes in rotational speed but also responds distinctly to subtle variations in tire deformation due to load. The consistency and resolution of the output signals across all scenarios affirm its effectiveness as a high-precision data acquisition node for smart tire platforms. Furthermore, experiments were performed on a closed road section, where the vehicle, subjected to a load of 3600 N, was driven over an identical distance at speeds of 30 km/h and 60 km/h, respectively. As presented in Figure 6c,d, the resistance variation of the sensor exhibits a pronounced reduction with increasing vehicle speed. Owing to the gravel nature of the test road surface, the sensor signals display non-uniform peak responses, thereby indicating that the proposed sensor possesses the capability to provide a preliminary assessment of road surface roughness, which is shown in Figure 6c,d.

In summary, the real-vehicle testing results comprehensively validate the sensor’s performance across multiple critical metrics, including sensitivity, stability, dynamic responsiveness, signal integrity, and environmental resilience. These results substantiate the sensor’s robust performance in both structured and unstructured driving environments, withstanding real-world mechanical, thermal, and vibrational disturbances. Given its outstanding characteristics, the LIG–Ecoflex piezoresistive sensor holds strong potential for multifunctional integration into next-generation intelligent transportation systems. Specifically, it can serve critical roles in the following fields: real-time vehicle health monitoring, detecting anomalies in tire behavior and load distribution; adaptive load estimation, improving ride comfort and dynamic control; road condition assessment, identifying rough or slippery surfaces to inform active safety systems; and early warning systems, enabling proactive responses to dangerous driving conditions such as sudden braking, loss of traction, or excessive tire stress. These capabilities make the proposed sensor a valuable enabler for the evolution of connected, intelligent, and safe vehicular platforms in the era of autonomous and electric mobility.

## 4. Conclusions

In this study, we developed and validated a flexible piezoresistive strain sensor based on laser-induced graphene embedded in an Ecoflex elastomer, with a focus on smart tire applications. The sensor exhibits high sensitivity (GF = 9.7), fast response (~255 ms), and stable electromechanical performance over 10,000 loading cycles. Owing to its anisotropic LIG microstructure, the device demonstrates strong directional recognition capabilities when coupled with a convolutional neural network, achieving a classification accuracy above 98%. Importantly, real-vehicle testing confirmed that the sensor reliably tracks tire deformation under varying loads and speeds, showing clear potential for practical automotive integration. The novelty of this work lies in combining direction-aware sensing with real-world tire validation, which bridges a critical gap between laboratory prototypes and practical deployment. Nonetheless, challenges remain, including long-term durability under harsh thermal and environmental conditions, as well as large-scale manufacturing consistency. Future studies will focus on multi-functional integration, such as the simultaneous monitoring of temperature, humidity, and vibration, along with wireless communication for real-time data fusion within IoV frameworks. Overall, this work demonstrates that LIG–Ecoflex-based sensors are promising candidates for next-generation intelligent tire systems, with broader applications in wearable devices, soft robotics, and structural health monitoring.

## Data Availability

The original contributions presented in this study are included in the article. Further inquiries can be directed to the corresponding authors.
